# TS-IBD: An efficient ancestral recombination graph-based identity by descent segment detection method

**DOI:** 10.47248/hpgg2606030009

**Published:** 2026-09-02

**Authors:** Yuan Wei, Ahsan Sanaullah, Degui Zhi, Shaojie Zhang

**Affiliations:** 1. Department of Computer Science, University of Central Florida, Orlando, FL 32816, USA; 2. McWilliams School of Biomedical Informatics, University of Texas Health Science Center at Houston, Houston, TX 77030, USA

**Keywords:** ancestral recombination graph, population genetics, identity by descent, tree sequence

## Abstract

The ancestral recombination graph (ARG) provides a comprehensive framework for representing the evolutionary history of genome sequences. Advances in ARG inference have enabled the extraction of informative, low-dimensional genomic features, such as identity by descent (IBD) segments, which are continuous genomic intervals remaining uninterrupted by recombination events. Extracting IBD segments requires explicit ARGs, and developing efficient algorithms for this purpose remains an active area of research. Here, we introduce TS-IBD, an efficient method that leverages the tree sequence formalism of ARGs to extract recombination-based IBD segments. TS-IBD is optimized for detecting short IBD segments and for use in memory-constrained environments. We show that IBD segments inferred by TS-IBD exhibit threefold lower inflation than those derived from genotype-based methods when analyzing IBD segment coverage in centromeric regions. Additionally, we compare our results with IBD segments inferred by alternative methods, showing that in simulated datasets with ground-truth ARGs, TS-IBD captures more recombination events in distant admixture than other methods. The TS-IBD program is available at https://github.com/ucfcbb/TS-IBD.

## 1. Introduction

Identity by descent (IBD) segments are shared genomic segments between a pair of individual haplotypes inherited from a common ancestor without recombination [1]. They are the groundwork for various genetic applications as they include information about genealogical connections between individuals. Studies have shown that IBD segments are considered useful for demographic history inference [2], genetic association mapping [3], and natural selection signal investigation [4]. Several IBD segment detection methods were developed, which were able to extract IBD segments efficiently from a genotype dataset [5]. However, IBD segments inferred from these genotype data may be erroneous due to the fact that inferring recombination breakpoint information directly from genotype data is challenging. Most of the methods are sequence comparison-based, and the called IBD segments may be mislabeled as identity by state (IBS) segments (i.e., having the same nucleotide sequence but not inherited from the same common ancestors). Chiang et al. investigated the identifiability problem of IBD segments, which is that the long IBD segments may arise from the conflation of smaller, nearby IBD segments [6]. The outcome may bias the length distribution of IBD segments and thus affect the results of certain downstream analyses.

Although the concept of ancestral recombination graph (ARG) was proposed long ago, it has recently become popular due to the availability of data and new methods [7]. Several ARG inference methods were developed to efficiently infer the ARG from a set of sequenced or genotyped genomes: RENT+ [8,9], ARGweaver [10,11], Relate [12], tsinfer [13], tcPBWT [14], ARG-Needle [15], and SINGER [16]. ARG incorporates the full evolutionary process of a group of individuals, capturing all recombination and coalescent events across the genome. Because ARG provides the entire evolutionary record, IBD segments inferred from it can achieve higher resolution than those inferred from genotype data. Thus, when the underlying ARG is of high quality and reflects the ground truth, ARG-derived IBD segment extraction provides a more robust and reliable approach than genotype-based methods.

With the emergence of high-quality and scalable ARG inference methods, several ARG-derived IBD segment extraction methods have been developed. Browning et al. [17] developed a most recent common ancestor (MRCA) based IBD segment detection method from simulated ancestry trees to have the extracted MRCA-based IBD segments as the ground truth for benchmarking the genotype data-derived MRCA-based IBD segment detection methods. The runtime of their approach is quadratic in the number of individuals in the sample, which limits the evaluation of all ancestry trees. Their method only samples the tree at fixed genetic distances (i.e., every five kilobases (kb) or 0.005 centiMorgan (cM)), and the extracted MRCA-based IBD segments may contain minor errors due to not evaluating the trees between every two sampled locations. Later, Yang et al. [18] proposed an MRCA-based IBD segment detection algorithm based on intersecting corresponding internal nodes (i.e., ancestors) between two trees to find pairs of leaf nodes (i.e., sampled individual haplotypes) with unchanged MRCA nodes. A candidate MRCA-based IBD segment covering the two trees is formed for those pairs. Then, the algorithm attempts to extend each pair’s candidate MRCA-based IBD segment by evaluating if the pair’s MRCA node remains unchanged on the chromosome in both directions. Thus, its time complexity is proportional to the square of the number of sampled individual haplotypes. Recently, Guo et al. implemented an MRCA-based IBD segment extraction method named tskibd [19] on top of the tskit library [20] and made it publicly available. The tskibd method adopts Browning et al.’s pairwise approach by comparing the MRCA of pairs of sampled individuals across all trees. Thus, its runtime is bounded quadratically in the number of sampled individual haplotypes. The tskit library [20] itself offers a preliminary function named “TreeSequence.ibd_segments()” to extract recombination-based IBD segments. We will refer to it as the tskit Python API method in the remainder of the paper. The tskit Python API is optimized for generating IBD summary statistics, not for extracting the complete set of IBD segments. It uses an approach to compute recombination-based IBD segments similar to how it constructs the tree sequence. However, as the tree sequence grows larger and the number of recombination-based IBD segments to be stored and output increases, the process can become more time-consuming and memory-intensive.

In addition to computational challenges, ARG-derived IBD segments are subject to definitional ambiguity, as different methods interpret them within the ARG in different ways. Most ARG-derived IBD segment inference methods adopt the MRCA-based definition, that is, an IBD segment is “a genomic segment between a pair of individual haplotypes for which the MRCA of the pair remains constant” [17]. This definition does not strictly follow the genetic definition of IBD as a chromosome segment: “two homologous chromosome segments are descended from a common ancestor without either of them experiencing a recombination” [21,22]. Alternatively, one can define IBD segments in an ARG by closely following the genetic definition: a genomic segment between a pair of individual haplotypes for which the segment has not been broken by any recombination events visible in the ARG. Though this recombination-based definition aligns more closely with the genetic concept of IBD, it remains relatively uncommon in practice, likely due to challenges in interpreting recombination breakpoints in an ARG. While the tskit Python API adopts this recombination-based IBD segment definition, it remains unclear how practical or effective the method is for IBD segment extraction across various scenarios. Challenges including scaling to large sample sizes or detecting very short IBD segments have not been addressed.

In this study, we introduce TS-IBD, an efficient algorithm for extracting recombination-based IBD segments from ARGs represented in the tree sequence format. TS-IBD has a substantially low growth rate of both runtime and memory with respect to the size of the tree sequence and the number of detected IBD segments. Our results show that TS-IBD outperforms the existing method for inferring short IBD segments, using less runtime and memory and scaling well to a large number of samples. Additionally, IBD segments inferred by TS-IBD from a high-quality or ground-truth ARG are reliable for IBD coverage analysis, as they result in substantially lower inflation than those inferred from genotype data in the centromeric region. Our analysis showed that the IBD segments generated by TS-IBD encode more recombination information on distant admixture than those from other methods. Overall, these strengths make TS-IBD a valuable IBD segment extraction method for a wide range of population genetic analyses.

## 2. Materials and Methods

### 2.1. Definition of ancestral recombination graph and tree sequence

Ancestral recombination graph, first defined by Griffiths et al. [23], is a directed acyclic graph containing a full genealogical history of a set of genome sequences. ARG was developed based on the coalescent theory [24,25], which assumes genome samples originated from a common ancestor and observes the populations backward in time. The ancestry of any pair of samples can be traced back to where they coalesce in their MRCA. The ARG offers a comprehensive representation of the coalescent process underlying the sampled genome sequences, while remaining concise as a graph that involves only nodes and edges corresponding to recombination and mutation events.

One of the efficient representations of an ARG is a sequence of correlated genetic trees located at different positions along a chromosome, i.e., a tree sequence [26]. It is a graph composed of nodes, edges, and associated metadata. Formally, it is defined as $T=\{G^{1},\ldots ,G^{L}\}$, a set of L local trees covering all positions of the genome. A local tree, $G^{x}$, in a tree sequence T is defined as $G^{x}=(V^{x},E^{x})$, where x is the index of local trees. $G^{x}$ describes relationships of individual haplotypes covering the genome positions $\left[l^{x},r^{x}\right)$. For a tree sequence having L local trees, x is in the range of [1, L]. $r^{x}=l^{x+1}$, $l_{1}$ is the start of the genome, and $r_{L}$ is the end of the genome. Assuming there are *n* nodes in $G^{x}$, $V^{x}=\{u_{1}^{x},u_{2}^{x},\cdots ,u_{n}^{x}\}$ is a sequence of integers representing the node IDs in $G^{x}$. The value of node ID is from the range [1,∣V∣]. Each node represents an individual haplotype and its node ID stays the same in the tree sequence. The set of leaf nodes does not change across local trees, but the set of internal nodes may vary in each local tree, depending on the tree’s topology. All ancestor nodes in the set of internal nodes of each leaf node are present in each local tree if the leaf nodes inherit the genome segment from the ancestor nodes in the local tree. Both leaf and internal nodes are in $V^{x}$. Following the genome ARG definition that Wong et al. proposed [27], $\left\{\pi_{1}^{x},\pi_{2}^{x},\cdots ,\pi_{n}^{x}\right\}$ is a sequence of integers, where $\pi_{u}^{x}$ denotes the parent node of *u*, and $\pi_{u}^{x}=0$ if *u* is a root node. $E^{x}=\{(u,\pi_{u}^{x},\{x\})\;|\;\pi_{u}^{x}\neq 0\}$ is a set of edges. The consistency of genealogies presented in local trees is preserved such that if *u* is a descendant of *v*, *u* is in a sub-tree of *v* in at least one of the local trees. *v* resides on the path from the root node to *u* in $G^{x}$, iff *u* inherits the genome positions $\left[l^{x},r^{x}\right)$ from *v*.

### 2.2. Definitions of identity by descent segment

**Figure 1.**
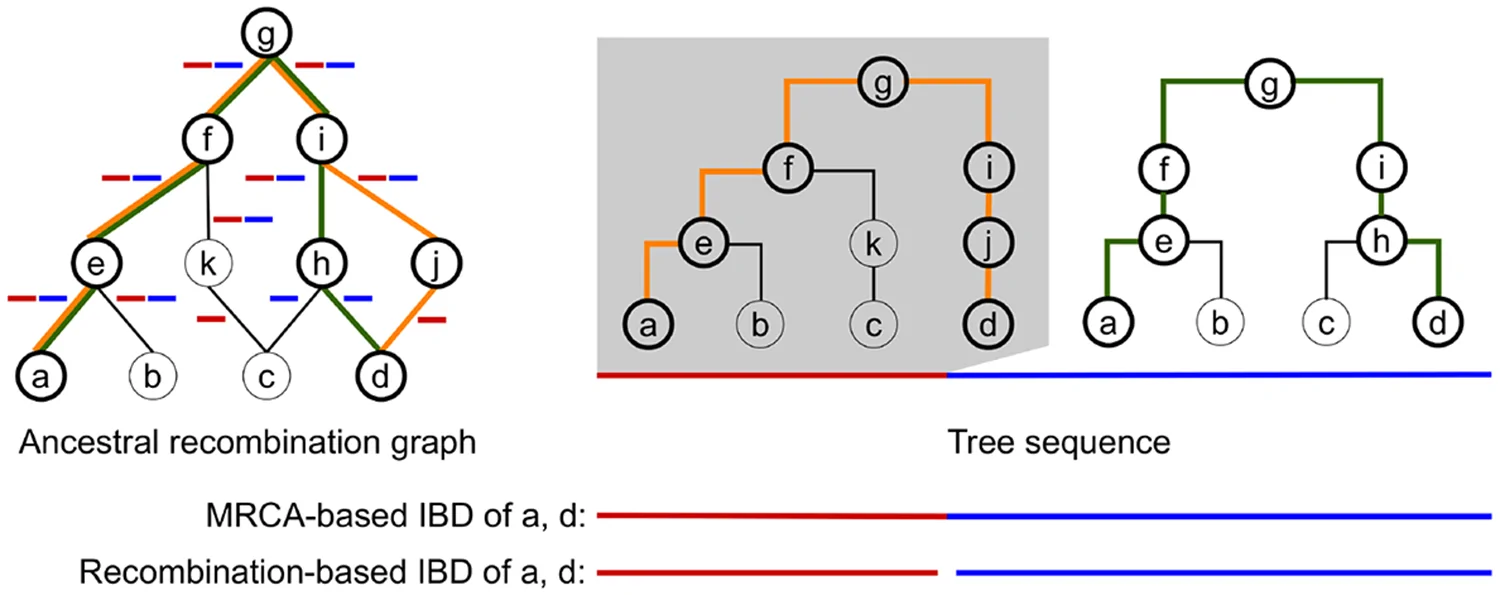
An example demonstrates the difference between the two definitions of inferred IBD segments from an ARG. Two segments (in red and blue) passed from an ancestor node *g* are illustrated in both the ancestral recombination graph (left) and the tree sequence (right). The recombination-based IBD and MRCA-based IBD segments of individual pairs *a* and *d* are different due to their definitions.

In a ground truth ARG where all current and past individuals are represented, recombination-based IBD segments can capture all recombination breakpoints among individuals. While some recombination events may not be visible on the ARG depending on its quality, the recombination-based IBD segments derived from an ARG inferred from a set of genotyped genomes are generally a better option than the MRCA-based IBD segments, as they may correspond more closely to the actual recombination events. Recombination-based IBD segments are increasingly favored for downstream analyses [28]. Figure 1 is an example demonstrating the difference between the two definitions of inferred IBD segments from an ARG. Individual pair *a* and *d* share different IBD segments depending on which definition is used. In the ancestral recombination graph, a red segment and a blue segment are passed from an ancestor node *g*. The same segments are presented in the tree sequence format, and each tree represents a segment. While one MRCA-based IBD segment is reported between individual pairs *a* and *d*, two recombination-based IBD segments are reported, as the definition of recombination-based IBD segment captures the recombination event caused by *h* and *j* on *d*.

From a genetic perspective, an identity by descent segment can be defined as a genomic segment between a pair of individual haplotypes which has been passed to the individuals unbroken by recombination from a common ancestor. In the ancestral recombination graph and tree sequence perspective, this is equivalently defined as a segment for which the MRCA of the pair remains constant, the entire segment has been inherited along the same genealogical path, and the segment cannot be further extended from either end. Thus, a recombination-based IBD segment of a pair of nodes *u* and *v* with a start and an end position $\left[l^{x_{1}},r^{x_{2}}\right)$ from local tree $x_{1}$ to $x_{2}\;(x_{1}\leq x_{2})$ in T can be defined as a tuple $\left(u,v,l^{x_{1}},r^{x_{2}}\right)$ such that an ancestral node *a* is the MRCA of node *u* and *v* from local trees $x_{1}$ to $x_{2}$ for the segment $\left[l^{x_{1}},r^{x_{2}}\right)$, and the ordered sequence of nodes for the path from *u* to *a* is the same, and the ordered sequence of nodes for the path from *v* to *a* is the same in all trees within the segment $\left[l^{x_{1}},r^{x_{2}}\right)$. The segment is a maximally contiguous genomic region where the ordered sequence of nodes for the path from *u* to *a* is the same, and the ordered sequence of nodes for the path from *v* to *a* is the same, and the MRCA (*a*) of *u* and *v* remains unchanged.

Less restrictive than the definition of the recombination-based IBD segment, a MRCA-based IBD segment (also can be described as identity by indirect descent) can be defined as a genomic segment between a pair of individual haplotypes for which the MRCA of the pair remains constant and the segment cannot be further extended from either end. The formal definition of a MRCA-based IBD segment of a pair of nodes *u* and *v* with a start and an end position $\left[l^{x_{1}},r^{x_{2}}\right)$ from local tree $x_{1}$ to $x_{2}\;(x_{1}\leq x_{2})$ in T is a tuple $\left(u,v,l^{x_{1}},r^{x_{2}}\right)$ such that an ancestral node *a* is the MRCA of node *u* and *v* from local trees *x*_1_ to *x*_2_ for the segment $\left[l^{x_{1}},r^{x_{2}}\right)$, and the segment $\left[l^{x_{1}},r^{x_{2}}\right)$ is a maximally contiguous genomic region where the MRCA (*a*) of *u* and *v* remains unchanged.

Assuming there are *m* leaf nodes $S=\{u_{1},u_{2},\cdots ,u_{m}\}$ in each local tree representing the sampled individual haplotypes at the current generation (*x* is dropped here and in the following for ease of notation since each local tree contains the same set of leaf nodes), the problem of finding all IBD segments between each pair of sampled individual haplotypes in a given tree sequence can be defined as a process to extract a set of IBD segments {(u,v,l,r)∣u,v∈S} from T.

### 2.3. From tree sequence to identity by descent segments

We propose a tree sequence identity by descent (TS-IBD) algorithm to infer recombination-based IBD segments from a tree sequence. The algorithm is based on subtree-prune-and-regraft (SPR) operations, iteratively traversing all local trees according to their genome positions and identifying topology changes. The topology changes regarding leaf nodes between two adjacent local trees are identified and recorded in a difference matrix. Then, adopting the positional Burrows–Wheeler transform (PBWT) algorithm [29], recombination-based IBD segments are identified from a list of difference matrices from the tree sequence. The PBWT algorithm is designed to efficiently index and identify matches on a large set of genetic sequences. In a panel containing *M* individual haplotypes and *N* genetic loci, the algorithm sorts haplotype sequences by their reversed prefixes at each locus. This approach enables all *C* matches among the haplotypes to be found in time complexity O(NM+C). Though the PBWT algorithm has shown its efficiency and has been used in many approaches, most of them store haplotype allele values in a PBWT data structure, such as tcPBWT; TS-IBD stores a sequence of topology change indicators.

To identify the topology changes regarding leaf nodes between tree $G^{x}$ and $G^{x+1}$, for a node $u\in G^{x}$, any leaf nodes under *u* are recorded in the difference matrix $D^{x,x+1}$ if *u* is not in $G^{x+1}$, or if *u* is in $G^{x+1}$ but its parent node in $G^{x}$ has changed in $G^{x+1}$. A difference matrix $D^{x,x+1}$ is a collection of topology change indicators for all leaf nodes between $G^{x}$ and $G^{x+1}$. Each row in $D^{x,x+1}$ corresponds to a leaf node and the topology change indicators for the leaf node. The topology change indicator is in two mutually exclusive states, 0 or 1. If *G^x^*’s topology is changed due to a node’s change (i.e., the node is pruned or regrafted), the topology change indicators in $D^{x,x+1}$ corresponding to those leaf nodes in the subtree of such node are set to 1; otherwise 0. Once all topology changes are identified and recorded in $D^{x,x+1}$, the PBWT algorithm is applied to $D^{x,x+1}$ to locate IBD segments. For leaf nodes in $D^{x,x+1}$, a match is considered only if all positions’ topology change indicator values are identical.

Alternatively, the difference matrix $D^{x,x+1}$ can be considered as a collection of multi-allelic values of individual haplotypes for a genome location. Thus, all columns in $D^{x,x+1}$ can be collapsed as one. For example, each row in $D^{x,x+1}$ can be considered as a binary number and converted into a decimal number by calculating the sum of the powers of 2 represented by each 1 bit. Then, the multi-allelic PBWT algorithm [30] can be applied to infer recombination-based IBD segments. However, the set of possible multi-allelic values can be very large (up to $2^{|S|}$). The larger the alphabet size is, the less efficient the multi-allelic PBWT algorithm becomes. Therefore, our approach chooses to use the “binary” format for each difference matrix with the original PBWT algorithm to infer recombination-based IBD segments.

**Figure 2.**
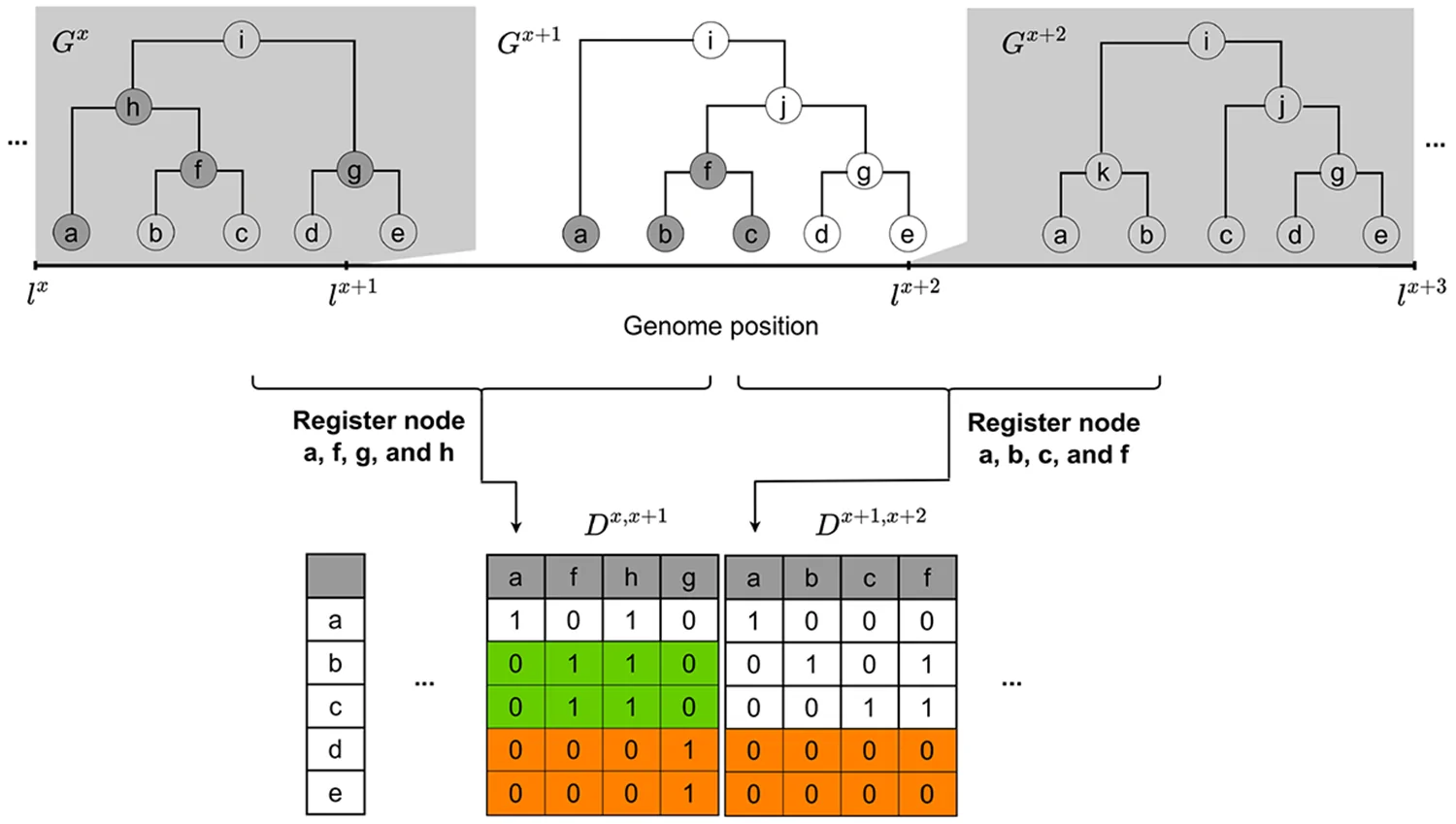
An example of recording the degree of the topology changes for the leaf nodes between tree $G^{x}$, $G^{x+1}$, and $G^{x+2}$. From $G^{x}$ to $G^{x+1}$, node *f* and its sub-tree is pruned from node *h* and regrafted to node *j*, which causes topology changes regarding nodes *a*, *f*, *h*, and *g* (shown in gray color). Similarly, from $G^{x+1}$ to $G^{x+2}$, node *b* is pruned from node *f* and regrafted to node *k*, causing topology changes regarding nodes *a*, *b*, *c*, and *f* (shown in gray color). They are registered as four columns in matrix $D^{x,x+1}$ and $D^{x+1,x+2}$ respectively, and the topology change indicators of the corresponding leaf nodes are set to 1. The recombination-based IBD segments (shown in green color for leaf node *b* and *c*, and orange color for leaf node *d* and *e*) are identified by applying the PBWT algorithm to the matrices.

Figure 2 is an example of recording the degree of the topology changes for the leaf nodes between tree $G^{x}$, $G^{x+1}$, and $G^{x+2}$ in the difference matrix $D^{x,x+1}$ and $D^{x+1,x+2}$. From $G^{x}$ to $G^{x+1}$, node *f* and its subtree is pruned from node *h* and regrafted to node *j*. This operation causes topology changes regarding nodes *a*, *f*, *h*, and *g*. They are registered as four columns in the difference matrix $D^{x,x+1}$ with the value 1 for topology change indicators corresponding to the leaf nodes in the subtree of those nodes. Since node *a* is a leaf node, $D^{x,x+1}[a][a]$ is set to 1. For internal nodes *f*, *h*, and *g*, the leaf nodes in their subtrees are set to 1 (i.e., $D^{x,x+1}[b][f],D^{x,x+1}[c][f],D^{x,x+1}[a][h],D^{x,x+1}[b][h],D^{x,x+1}[c][h]$, and $D^{x,x+1}[d][g],D^{x,x+1}[e][g]$ are set to 1). Similarly, from $G^{x+1}$ to $G^{x+2}$, node *b* is pruned from node *f* and regrafted to node *k*, causing topology changes regarding nodes *a*, *b*, *c*, and *f*, which are registered in $D^{x+1,x+2}$.

To infer recombination-based IBD segments between $G^{x}$ and $G^{x+1}$, the PBWT algorithm is applied to the difference matrix $D^{x,x+1}$. In this example, only two leaf nodes have a match: *c* matches *b*, and *e* matches *d*. For leaf node *b*, its corresponding sequence only matches the one of leaf node *a* partially (i.e., position *h* and *g*, but not position *a* or *f*). Thus, this match was discarded and *b* is not considered as a match of *a* in $D^{x,x+1}$. The match between *c* and *b* is ended in $D^{x,x+1}$, and the match between *e* and *d* is extended to $D^{x+1,x+2}$.

Algorithm 1 demonstrates how to identify the nodes affected by the topology changes from tree $G^{x}$ to $G^{x+1}$. From the implementation perspective, edge differences are evaluated to identify the topology changes between adjacent trees (this operation equals the steps from lines 2 to 3 in Algorithm 1). Since tree sequence stores all edges E in the form of (left interval, right interval, parent node, child node), and the genomic interval may span across multiple trees (implying that ℰ<<LE), an efficient way to identify edge differences is to sort edges by their right intervals and iterate over them alongside with trees. A different edge is identified if its right interval equals a local tree’s end position. Then, the child node of the edge is registered in matrix *D*. This approach takes O(∣ℰ∣log∣ℰ∣) for edge sorting and O(L+∣ℰ∣) for iteration, which is faster than evaluating all nodes in a tree (i.e., O(L(V+E))).

**Table:** 

Algorithm 1. Building the difference matrix.
$$\begin{array}{cc} \; & \;1:A^{x,x+1}=\emptyset ,\;D^{x,x+1}=\emptyset \\ \; & \;2:\mathrm{for}\;u\in V^{x}\;\mathrm{do}\\ \; & \;3:\;\;\mathrm{if}\;u\notin V^{x+1}\;\text{or}\;\pi_{u}^{x}\neq \pi_{u}^{x+1}\;\mathrm{then}\\ \; & \;4:\;\;\;\;COLLECT\_NODES(u)\\ \; & \;5:\;\;\;\;\mathrm{for}\;v\in S\;\mathrm{do}\\ \; & \;6:\;\;\;\;\;\;\mathrm{if}\;v\in A^{x,x+1}[u]\;\mathrm{then}\\ \; & \;7:\;\;\;\;\;\;\;\;D^{x,x+1}[v][u]=1\\ \; & \;8:\;\;\;\;\;\;\mathrm{else}\\ \; & \;9:\;\;\;\;\;\;\;\;D^{x,x+1}[v][u]=0\\ \; & \;10:\;\;\\ \; & \;11:\mathrm{function}\;COLLECT\_NODES(u)\\ \; & \;12:\;\;\mathrm{if}\;u\notin S\;\mathrm{then}\\ \; & \;13:\;\;\;\;\mathrm{for}\;v\;\text{in}\;\{v\;|\;\pi_{v}=u\}\;\mathrm{do}\\ \; & \;14:\;\;\;\;\;\;COLLECT\_NODES(v)\\ \; & \;15:\;\;\;\;\;\;\mathrm{if}\;v\in S\mathrm{then}\\ \; & \;16:\;\;\;\;\;\;\;\;A^{x,x+1}[u]=A^{x,x+1}[u]\cup \{v\}\\ \; & \;17:\;\;\;\;\;\;\mathrm{else}\\ \; & \;18:\;\;\;\;\;\;\;\;A^{x,x+1}[u]=A^{x,x+1}[u]\cup A^{x,x+1}[v]\\ \; & \;19:\;\;\mathrm{else}\\ \; & \;20:\;\;\;\;A^{x,x+1}[u]=\{u\} \end{array}$$

$A^{x,x+1}$ is a leaf nodes matrix whose entry stores a set of all leaf nodes under a node (i.e., $A^{x,x+1}[u]$ stores a set of leaf nodes under *u*). The leaf nodes under *u* are retrieved and stored only when *u* is identified as a node affected by the topology changes between $G^{x}$ and $G^{x+1}$. The *COLLECT_NODES*(*u*) function is used to retrieve all leaf nodes under *u*. It traverses down the sub-tree from *u* and stores leaf nodes in $A^{x,x+1}[u]$. The runtime complexity of this operation is $O(V^{x}+E^{x})$, as the worst-case scenario *u* is the root node of $G^{x}$. Then, the leaf nodes under *u* are registered in $D^{x,x+1}$. It takes O(K∣S∣) for the registration, assuming there are *K* topology changes identified. *K* is a property of tree sequences that reflects the underlying recombination rate. The tree sequence is a compact and efficient structure, as it captures and encodes the shared structure between adjacent trees [31]. Therefore, adjacent trees have very few differences and *K* remains relatively small. Lastly, the PBWT algorithm is applied on all difference matrices to infer recombination-based IBD segments. The motivation for using the PBWT algorithm is that it offers an efficient solution for storing and extending IBD segments that span many consecutive trees. This capability makes it suitable for handling long-range IBD segment detection in large-scale genomic data. The runtime is O(K∣S∣+C), where C is the number of inferred recombination-based IBD segments. In practice, C can become substantial for large datasets. The overall runtime complexity of the TS-IBD algorithm is O(∣ℰ∣log∣ℰ∣+KL(V+E)+C), assuming V and E are the maximum number of nodes and edges of a tree in the tree sequence.

The TS-IBD algorithm iterates through the tree sequence once, dynamically computing difference matrices as it evaluates each local tree. It simultaneously applies the PBWT algorithm to these matrices in a streaming manner to compute the recombination-based IBD segments. Only two local trees (size of 2(*V* + *E*)) and two matrices (size of 2*KS*) are required to be present in memory at the same time. Thus, its space complexity is bounded by O(KS+V+E). This on-the-fly strategy enhances computational efficiency and significantly reduces memory consumption.

The correctness of the TS-IBD algorithm is substantiated in Section S1. Specifically, Lemma S1 demonstrates that TS-IBD identifies all recombination-based IBD segments in a given tree sequence. Furthermore, empirical validation (see Table S1) confirms that TS-IBD and the tskit Python API produce the same number and length of IBD segments.

## 3. Results

### 3.1. Runtime and memory

The performance of the TS-IBD algorithm was evaluated on Chromosome 22 datasets simulated by the stdpopsim library [32,33]. The simulation engine was set as msprime [26,34-36], and the demographic model used was the out-of-Africa model [37]. The GRCh38 recombination rate map [38,39] was applied to the simulation. Five tree sequences were simulated with 10, 100, 1,000, 10,000, and 100,000 individual haplotypes. The TS-IBD algorithm was compared with the tskit Python API method, as it is the only other recombination-based IBD segment extraction method currently available. Experiments were conducted on a machine with an Intel Xeon Gold 5215 2.50 GHz processor and limited memory usage of 100 GB of RAM (except the 1-million-haplotype test case, limited to 300 GB of RAM). Short recombination-based IBD segments were inferred with the minimum physical cutoff length of 100,000 base pairs using both approaches. We use 100,000 base pairs (roughly 0.1 cM) as the IBD segment target length because the tskit Python API does not support using genetic distance, which is more commonly used in IBD analysis. All experiments were run five times, and the mean values were reported, excluding those taking more than a day to complete.

Table 1 shows the runtimes and memories of both approaches for various numbers of sampled individual haplotypes. TS-IBD was faster and used less memory in the case of extracting short IBD segments. The tskit Python API approach generally requires more runtime and memory for the same cases. For example, TS-IBD took 30 minutes to complete the 10,000-haplotype test cases, while the tskit Python API took 4.8 days and used 42 times more memory. For the 100,000-haplotype test case, the tskit Python API could not complete the run in five days.

To further evaluate scalability, we simulated a tree sequence with 1,000,000 haplotypes and applied both methods with a 2,000,000-base-pair (2 megabases, roughly 2 cM) cutoff to output IBD segments. TS-IBD completed the task in approximately 25 hours using 0.87 gigabytes of memory. In contrast, the tskit Python API failed to finish the task when it consumed 181.15 gigabytes of memory.

### 3.2. IBD coverage

To illustrate the advantages of using recombination-based IBD segments in downstream applications, an IBD coverage analysis similar to the one that the hap-IBD method’s authors did [40] was conducted. IBD coverage is defined as the sum of the weights calculated by the proportion of the region that is covered by the IBD segments. Each region is 1 megabase pair in length. For this analysis, a Chromosome 1 dataset with 500 individual haplotypes, including both the tree sequence and the genotype data, was simulated using the stdpopsim library [32,33] and the American admixture demographic model [41]. The recombination-based IBD segments were extracted from the simulated tree sequence using the TS-IBD method as the ground truth. Then, the tsinfer [13] method was used to generate the inferred tree sequence from the genotype data. The ARG-derived IBD coverage was computed using the recombination-based IBD segments extracted from the inferred tree sequence by the TS-IBD method, and the genotype-derived IBD coverage was computed using the IBS segments extracted from the genotype data in VCF format by the hap-IBD method [40]. The 2 cM IBD segment cutoff length was applied to all methods. We excluded the tskit Python API from the analysis because it lacks support for specifying the IBD segment cutoff length with genetic distance as the measurement unit. This limitation restricts its flexibility for analyses because genetic distance is the more commonly used measure of the IBD segment.

**Figure 3.**
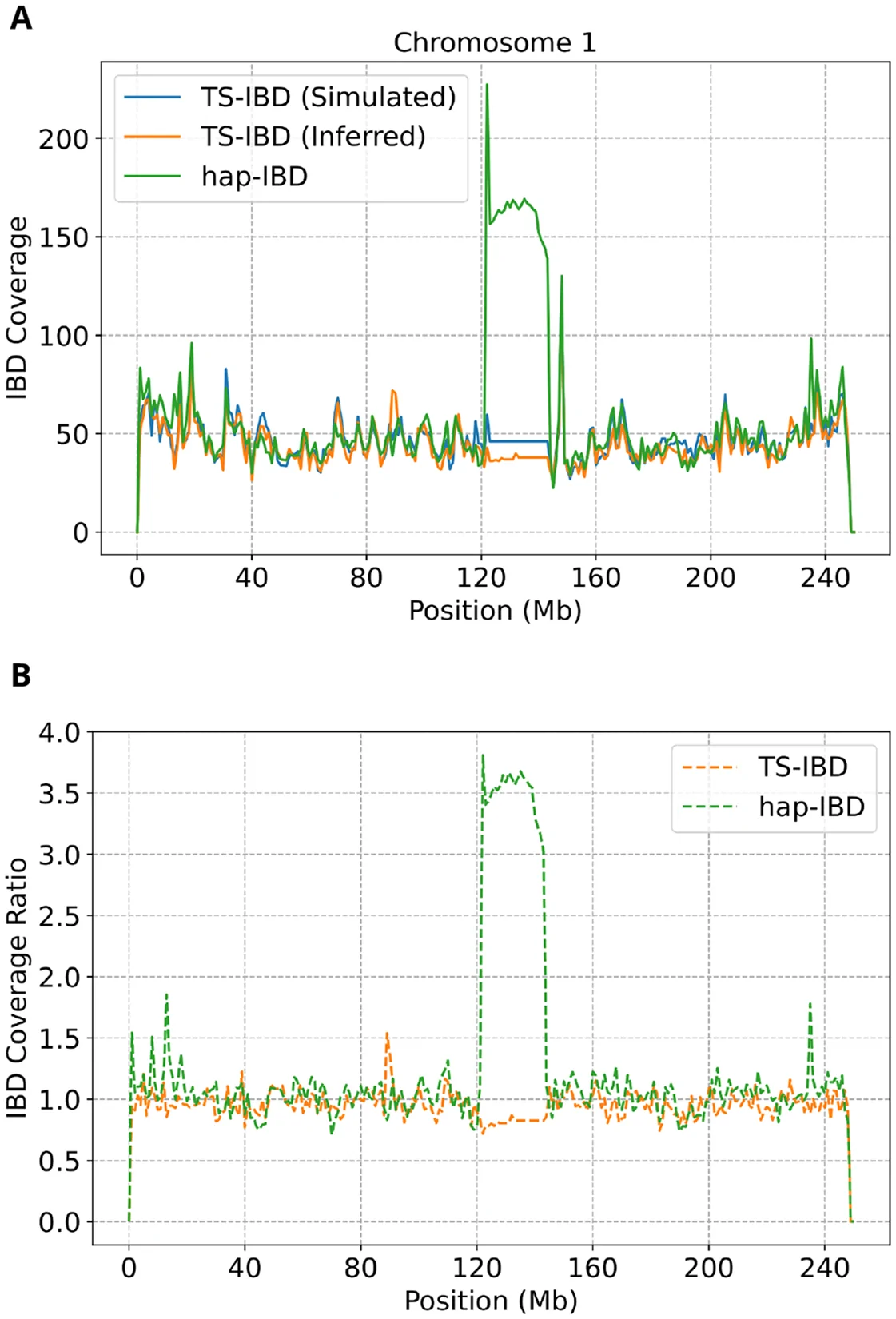
IBD coverage and ratio on Chromosome 1 calculated by ARG-derived recombination-based IBD segments (TS-IBD) on simulated and inferred ARGs, and genotype-derived IBS segments (hap-IBD) on simulated genotype dataset. (A) IBD coverage. (B) IBD coverage ratio (called IBD coverage divided by ground truth IBD coverage). The x-axis is the physical position in megabases. The bin size is 1 megabase pair, and the called IBD segment length is at least 2 cM.

Figure 3A shows that the genotype-derived IBD segments with a 2 cM cutoff length may have an inflation issue around the centromere and the pericentromere (1q12) of Chromosome 1. According to the report from the hap-IBD method’s authors, hap-IBD has the least impact on the inflation of IBD segments compared to other genotype-derived IBD segment calling methods [40]. However, the degree of inflation remains considerable. This inflation may be due to the sequence-comparison-based method used by hap-IBD for IBD segment detection. Because the centromeric region contains numerous repeated DNA sequences, the identified segments in this region may not represent true IBD segments, as the IBD segments are identified from the genotyping data, where their endpoints may not be the exact recombination breakpoints. In contrast, recombination-based IBD segments are free from the potential issue of identifying IBD segments from repeated DNA sequences. Their endpoints of IBD segments correspond to the genomic positions of tree intervals. In a high-quality inferred tree sequence, the genomic positions of tree intervals are very close to where recombination events take place.

Our results show that using TS-IBD yields lower inflation in the region, indicating that it could help reduce inflation while preserving the ability to infer short IBD segments. To measure the degree of inflation, IBD coverage ratios were calculated. It is defined as the called IBD coverage divided by the ground truth IBD coverage. Figure 3B illustrates that the hap-IBD called IBD coverage exhibits more than a threefold inflation in centromeric and pericentromeric regions compared to the ground truth. In contrast, the TS-IBD called IBD coverage does not display such elevated inflation levels in these regions.

### 3.3. Length distribution of shared IBD segments

IBD-driven methods have been extensively used to estimate various parameters in population demographic models, such as effective population size [42-44,28]. Since IBD segments are genome regions not broken by recombination events, the recombination-based IBD segments may be more useful than the MRCA-based IBD segments for population demographic inference, as they present a full view of recombination events in the population’s demographic history.

To illustrate the difference between IBD segments derived from different methods, we simulated a Chromosome 22 dataset with 500 individual haplotypes using msprime and the American admixture demographic model [41], including both the ground truth tree sequence and its genotype data form. We plotted the fraction of the genome shared through IBD segments. The fraction of the genome is calculated as the summation of all the IBD segments whose length is greater than or equal to the length bin size, divided by the product of the total genome length and the number of individual pairs. We included cases with IBD segments inferred by TS-IBD, by tskibd, and by hap-IBD in the calculations. We did not include the tskit Python API in this experiment because it is specialized for outputting IBD summary statistics. Using it for this analysis would be extremely time-consuming. In a previous test with 10,000 haplotypes, it took 4.8 days to extract 600 million IBD segments. In this experiment, TS-IBD output 5.7 billion recombination-based IBD segments. We estimate that extracting this many IBD segments with the tskit Python API would take approximately 46 days. We excluded large datasets from this experiment due to the substantial computational time required to run tskibd. It took more than four days for tskibd to complete its run on the 500-individual-haplotype dataset described above.

**Figure 4.**
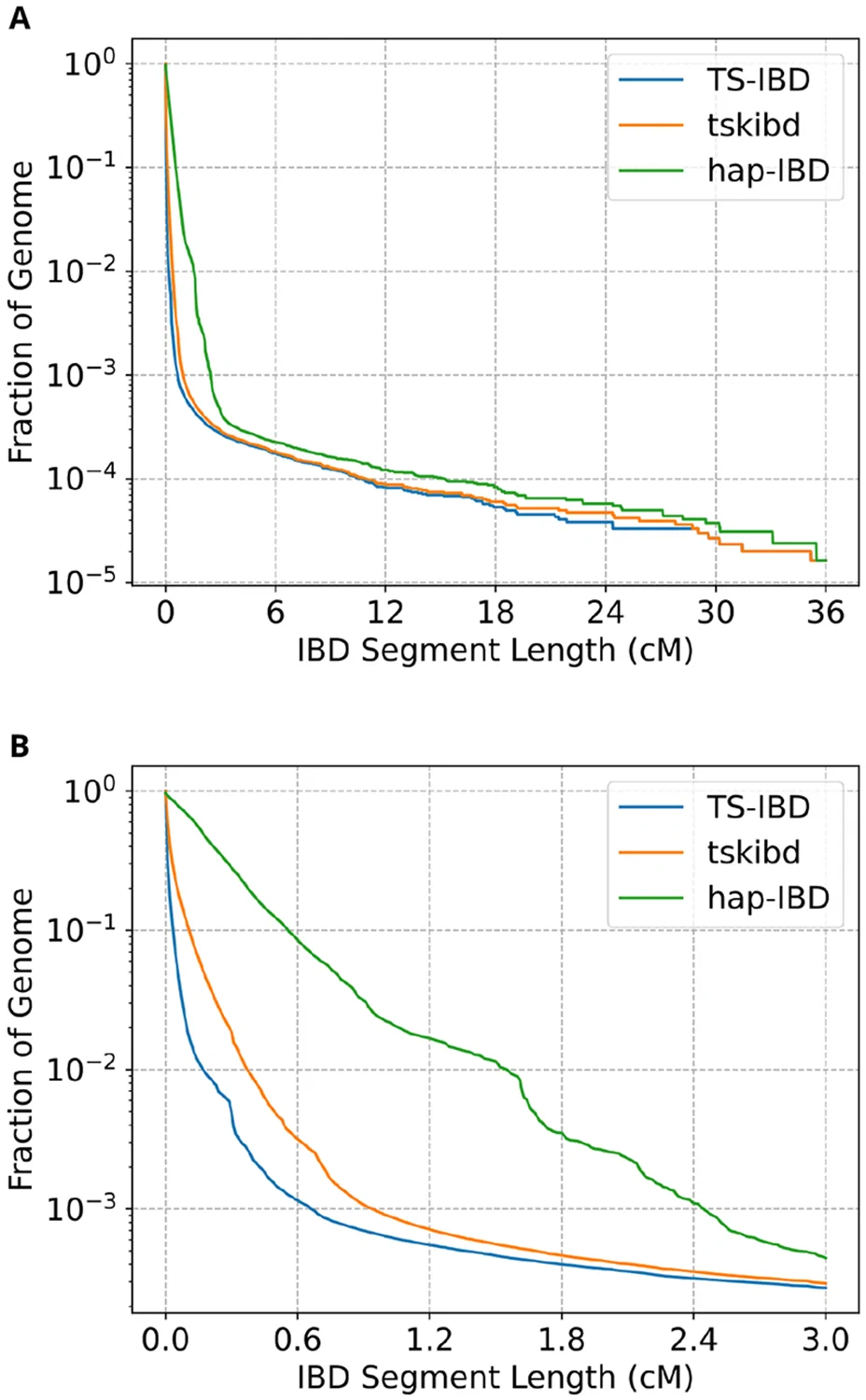
The fractions of the genome shared through TS-IBD, tskibd, and hap-IBD segments on a simulated tree sequence. (A) IBD segment length less than 36 cM. (B) IBD segment length less than 3 cM. Each IBD segment length bin size is 0.01 cM.

Figure 4A shows the fractions of genome shared through IBD segments up to 36 cM. Figure 4B is a zoomed-in version illustrating the fractions of the genome of the types of IBD segments within 3 cM. It is observed that IBD segments inferred from TS-IBD represent a relatively smaller fraction of the genome per length, compared to those from tskibd and hap-IBD, particularly when the length of the IBD segment is short (e.g., less than 3 cM). In other words, it takes a larger number of recombination-based IBD segments to cover the same fraction of the genome, compared to using MRCA-based IBD or IBS segments. Short IBD segments are associated with distant coalescent events because they result from recombinations occurring over many generations. Therefore, IBD segments inferred from TS-IBD may offer clearer insights into distant demographic history by including shorter IBD segments that capture more recombination events. However, additional validation is needed to confirm this benefit.

## 4. Discussion

We presented TS-IBD, an ARG-derived method that efficiently extracts recombination-based IBD segments from tree sequences. TS-IBD is optimized for extracting short IBD segments with feasible memory usage and allows users to specify the target IBD segment length in either physical or genetic distance. Our benchmarking demonstrated that TS-IBD generally outperforms the existing method in both runtime and memory usage for short IBD segment extraction. TS-IBD can scale to process large datasets, handling up to one million individual haplotypes. Our analyses showed that IBD segments inferred using TS-IBD from a high-quality or ground-truth ARG have lower inflation levels than those from the genotype-based method, and represent a relatively smaller fraction of the genome per length, potentially reflecting more recombination events. These advantages make TS-IBD a robust and practical tool for researchers seeking reliable IBD segments within the ARG framework when a high-quality or ground-truth ARG is available, facilitating diverse applications in population genetics.

TS-IBD serves as a powerful benchmarking tool for generating the ground truth of short recombination-based IBD segments from tree sequence-based data. Currently, it takes either physical or genetic distance as the cutoff length parameter for IBD segment filtering. Expanding its filtering capabilities to incorporate parameters such as time depth and targeted subsets of haplotypes would further increase its flexibility and effectiveness for complex analyses. While these IBD segments provide an accurate description of genealogical connections between individuals, the number of IBD segments can grow rapidly when identifying short segments in large biobank-scale datasets. Under the circumstances, model-based IBD segments generated from the approximate ARG may be preferable, as processing enormous numbers of IBD segments for downstream analysis can be impractical. Those IBD segments can either be estimated from a small subset of ARG-derived IBD segments [45], or simulated directly from coalescent modeling [46-49]. Typically, to simplify the process, statistical approaches produce fewer approximate IBD segments than exact IBD segments. However, using approximate IBD segments may result in a loss of accuracy, depending on the specific application. Sometimes, users are interested only in summary statistics (e.g., the total number of IBD segments) rather than outputting all IBD segments. The tskit Python API is designed to handle such tasks quickly, but at the expense of high memory consumption. Section S2 presents details of the IBD summary statistics experiments, with the corresponding results summarized in Table S2.

While IBD segments inferred by TS-IBD show promise in simulation studies, their application to real-world datasets remains challenging. Many ARG inference methods struggle to produce high-quality ARGs from genotyping data, thereby limiting the reliability of IBD segments inferred from those ARGs. A small difference between inferred ARGs can produce distinct tree sequences, each encoding a different number of recombination events and producing different sets of IBD segments. As illustrated in Section S3, a single differing internal node can result in different ARGs, leading to different numbers of recombination events and different sets of output IBD segments (see Figure S1 for an example). Nevertheless, as ARG inference methods continue to advance and achieve higher resolution, TS-IBD is poised to become an increasingly valuable tool for IBD-driven downstream analyses.

## Supplementary Material

Supplementary Materials

The following supplementary materials are available on the website of this paper: HPGG2606030009SupplementaryMaterials.zip.

1. Section S1. Correctness of the TS-IBD algorithm.
2. Section S2. Runtime and memory without output IBD segments.
3. Section S3. Examples of tree sequences and their corresponding ancestral recombination graphs.
4. Lemma S1. Given a tree sequence T, the TS-IBD algorithm outputs all and only recombination-based IBD segments.
5. Table S1. Total number and length of recombination-based IBD segments extracted using the TS-IBD algorithm (or the tskit Python API approach) with minimum physical length 100,000 base pairs from msprime-simulated Out-of-Africa Chromosome 22 datasets.
6. Table S2. Runtime (seconds) and memory (megabytes) of the summary of recombination-based IBD segments using the TS-IBD algorithm and the tskit Python API approach with minimum physical length 100,000 on msprime-simulated Out-of-Africa Chromosome 22 datasets.
7. Figure S1. Three tree sequence examples A, B, and C with their corresponding ARGs and SPR operations.

## Figures and Tables

**Table 1.** Runtime (seconds) and memory (megabytes) of extracting recombination-based IBD segments using TS-IBD and tskit Python API, with minimum physical length 100,000 on msprime-simulated Out-of-Africa Chromosome 22 datasets. Results are the means from five independent runs, except for tasks taking more than a day to complete; in these cases, results from a single run are reported.

TS-IBD			tskit Python API	
Number of Haplotypes	Runtime	Memory	Runtime	Memory
10	0.35	50.95	1.61	113.95
100	2.72	120.95	46.78	232.37
1,000	49.30	274.20	3,616.91	515.59
10,000	1,823.40	573.78	414,916.80	24,112.90
100,000	116,858.00	1,046.04	na	na
