# Supplementary material for: TS-IBD: An efficient ancestral recombination graph-based identity by descent segment detection method
Source: Hum Popul Genet Genom. Author manuscript; Available in PMC 2026 Sep 22. (PMC13591471; doi:10.47248/hpgg2606030009)
Supplement: Supplementary file 1 [file NIHMS2209121-supplement-Supplementary_Materials.pdf]

Human Population Genetics and Genomics
https://doi.org/10.47248/hpgg2606030009

Original Research

TS-IBD: An efficient ancestral recombination graph-based identity by descent segment detection method

Yuan Wei [1], Ahsan Sanaullah [1], Degui Zhi [2], Shaojie Zhang [1,*]

1. Department of Computer Science, University of Central Florida, Orlando, FL 32816, USA; Emails: Yuan.Wei@ucf.edu (Y.W.); Ahsan.Sanaullah@ucf.edu (A.S.)
2. McWilliams School of Biomedical Informatics, University of Texas Health Science Center at Houston, Houston, TX 77030, USA; Email: Degui.Zhi@uth.tmc.edu

* **Correspondence:** Shaojie Zhang; Email: shzhang@cs.ucf.edu

Supplementary Materials

S1. Correctness of the TS-IBD algorithm

The TS-IBD algorithm is demonstrated to correctly produce all recombination-based IBD segments from a given tree sequence, as shown in the following lemma.

Lemma S1. *Given a tree sequence T, the TS-IBD algorithm outputs all and only recombination-based IBD segments.*

Proof. $\forall\, a, b \in S$ in G^x, there exists a unique path $P^x_{ab} = (u_1, u_2, \ldots, u_k)$, which is an ordered sequence of nodes that $u_1 = a$, $u_k = b$, $u_{i+1} = \pi_{u_i}$ or $u_i = \pi_{u_{i+1}}$, $u_i \in V^x$, $i, j, k \in [1, |V^x|]$, and $\forall\, i \neq j$, $u_i \neq u_j$. The MRCA node of a and b in G^x is $MRCA(a, b, x) = u_i$, where u_i is in P^x_{ab} and $u_i = \pi_{u_{i-1}}$ and $u_i = \pi_{u_{i+1}}$. Let $P^x_{ab} = (u_1, u_2, \ldots, u_{k_1})$ be the path in G^x, and $P^{x+1}_{ab} = (v_1, v_2, \ldots, v_{k_2})$ be the path in G^{x+1}. Based on the definition of a recombination-based IBD segment, the boundary of recombination-based IBD segments of a and b is identified between G^x and G^{x+1} if $MRCA(a, b, x) \neq MRCA(a, b, x+1)$, or $k_1 \neq k_2$ or $\exists\, i \leq min(k_1, k_2)$, $u_i \neq v_i$. This implies $P^x_{ab} \neq P^{x+1}_{ab}$, where $\exists\, u$ in P^x_{ab}, $u \neq MRCA(a, b, x)$, $u \notin V^{x+1}$ or $\pi^x_u \neq \pi^{x+1}_u$, and exactly one of the nodes in $\{a, b\}$ belongs to the subtree rooted at u in G^x. The TS-IBD algorithm records the node in the subtree of u as 1 in $D^{x,x+1}$ and 0 otherwise (i.e., $D^{x,x+1}[a] \neq D^{x,x+1}[b]$). The reverse implication also holds since $D^{x,x+1}[a] \neq D^{x,x+1}[b]$ implies that exactly one of the nodes in $\{a, b\}$ belongs to the subtree rooted at u in G^x where u in P^x_{ab}, $u \neq MRCA(a, b, x)$, and $u \notin V^{x+1}$ or $\pi^x_u \neq \pi^{x+1}_u$; hence $P^x_{ab} \neq P^{x+1}_{ab}$. Let $D^{x,\ldots,y}$ be the concatenation of the consecutive difference matrices from $D^{x,x+1}$ through $D^{y-1,y}$, i.e., $D^{x,\ldots,y}[a] = D^{x,\ldots,y}[b] \iff \forall\, z \in \{x, \ldots, y-1\}$, $D^{z,z+1}[a] = D^{z,z+1}[b]$. TS-IBD applies PBWT algorithm to report maximal matching intervals $D^{x,\ldots,y}[u_i] = D^{x,\ldots,y}[u_j]$ between sample nodes $u_i, u_j \in S$ for $x, y \in [1, L]$ in $T = \{G^1, \ldots, G^L\}$. $D^{x,\ldots,y}[u_i] = D^{x,\ldots,y}[u_j]$ means the paths between u_i and u_j remain unchanged throughout the interval $[x, y)$, and it cannot be extended to either side because $D^{x-1,x}[u_i] \neq D^{x-1,x}[u_j]$ and $D^{y,y+1}[u_i] \neq D^{y,y+1}[u_j]$. Therefore, the corresponding match (u_i, u_j, l^x, r^y) is a maximal recombination-based IBD segment. Since TS-IBD evaluates all adjacent tree pairs in T for all sample nodes, it outputs all and only recombination-based IBD segments from T.

Empirical evidence confirms that TS-IBD and the tskit Python API produce the same set of IBD segments when using the same cutoff length. The total number and length of IBD segments (see Table S1) extracted from msprime-simulated Out-of-Africa Chromosome 22 datasets with minimum physical length 100,000 base pairs were recorded using both programs, and the values matched. The output IBD segments from the 10-haplotype case by both programs were compared, and they are exactly the same.

Table S1. Total number and length of recombination-based IBD segments extracted using the TS-IBD algorithm (or the tskit Python API approach) with minimum physical length 100,000 base pairs from msprime-simulated Out-of-Africa Chromosome 22 datasets.

Number of Haplotypes	Number of IBDs	Length of IBDs
10	859	156,733,829
100	70,243	11,372,097,179
1,000	5,522,016	912,765,581,280
10,000	600,360,869	93,814,325,220,411

S2. Runtime and memory without output IBD segments

The tskit Python API [1] is designed for getting IBD summary statistics. It has a built-in option not to output any IBD segments. This option streamlines data processing by minimizing unnecessary I/O operations when only a summary of IBD segments (e.g., the total number and length of IBDs) is needed. We implemented the summary-only option in the TS-IBD program and performed runtime and memory analysis on Chromosome 22 datasets simulated by the stdpopsim library [2,3] and the out-of-Africa model [4]. Five tree sequences were simulated with 10, 100, 1,000, 10,000, and 100,000 individual haplotypes. Experiments with a minimum physical cutoff length of 100,000 base pairs were conducted on a machine with an Intel Xeon Gold 5215 2.50 GHz processor and limited memory usage of 100 GB of RAM. All experiments were run five times, and the mean values were reported.

Table S2 shows the runtimes and memories of TS-IBD and tskit Python API for various numbers of sampled individual haplotypes. The tskit Python API is generally faster than TS-IBD when only the IBD summary is required. However, it comes at the cost of increased memory usage, often requiring substantially more memory to complete the task.

Table S2. Runtime (seconds) and memory (megabytes) of the summary of recombination-based IBD segments using the TS-IBD algorithm and the tskit Python API approach with minimum physical length 100,000 on msprime-simulated Out-of-Africa Chromosome 22 datasets.

		TS-IBD (Summary)		tskit Python API (Summary)	
Number of Haplotypes	Number of IBDs	Runtime	Memory	Runtime	Memory
10	859	0.36	50.85	0.97	113.92
100	70,243	2.56	121.11	1.51	232.50
1,000	5,522,016	42.40	273.66	3.42	486.62
10,000	600,360,869	1,003.45	574.51	17.45	985.19
100,000	59,856,097,080	47,539.20	1038.27	768.38	4,536.68

S3. Examples of tree sequences and their corresponding ancestral recombination graphs

There is a one-to-one correspondence between an Ancestral Recombination Graph (ARG) and the tree sequence it encodes [5]. Based on the given definition of recombination-based identity by descent (IBD) segment, IBD segments are explicitly determined from a tree sequence. Altering just one node in a tree sequence can dramatically change the resulting ARG. Figure S1 is an example with three tree sequences having subtle distinctions. They represent different ARGs and thus encode different numbers of recombinations and sets of recombination-based IBD segments. For instance, a pair of nodes c and d shares a recombination-based IBD segment $[0, 40)$ in cases B and C, but not case A.

A

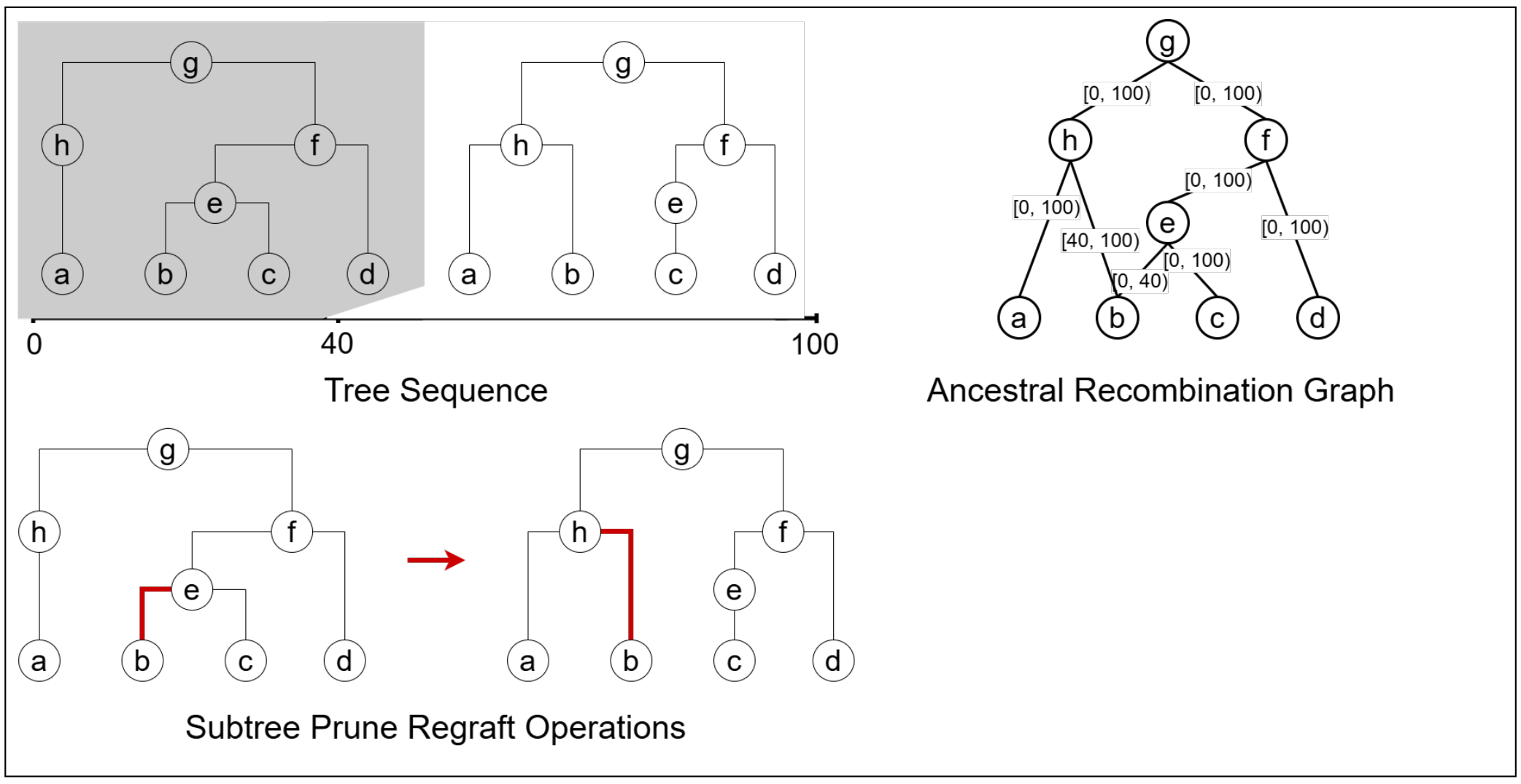

B

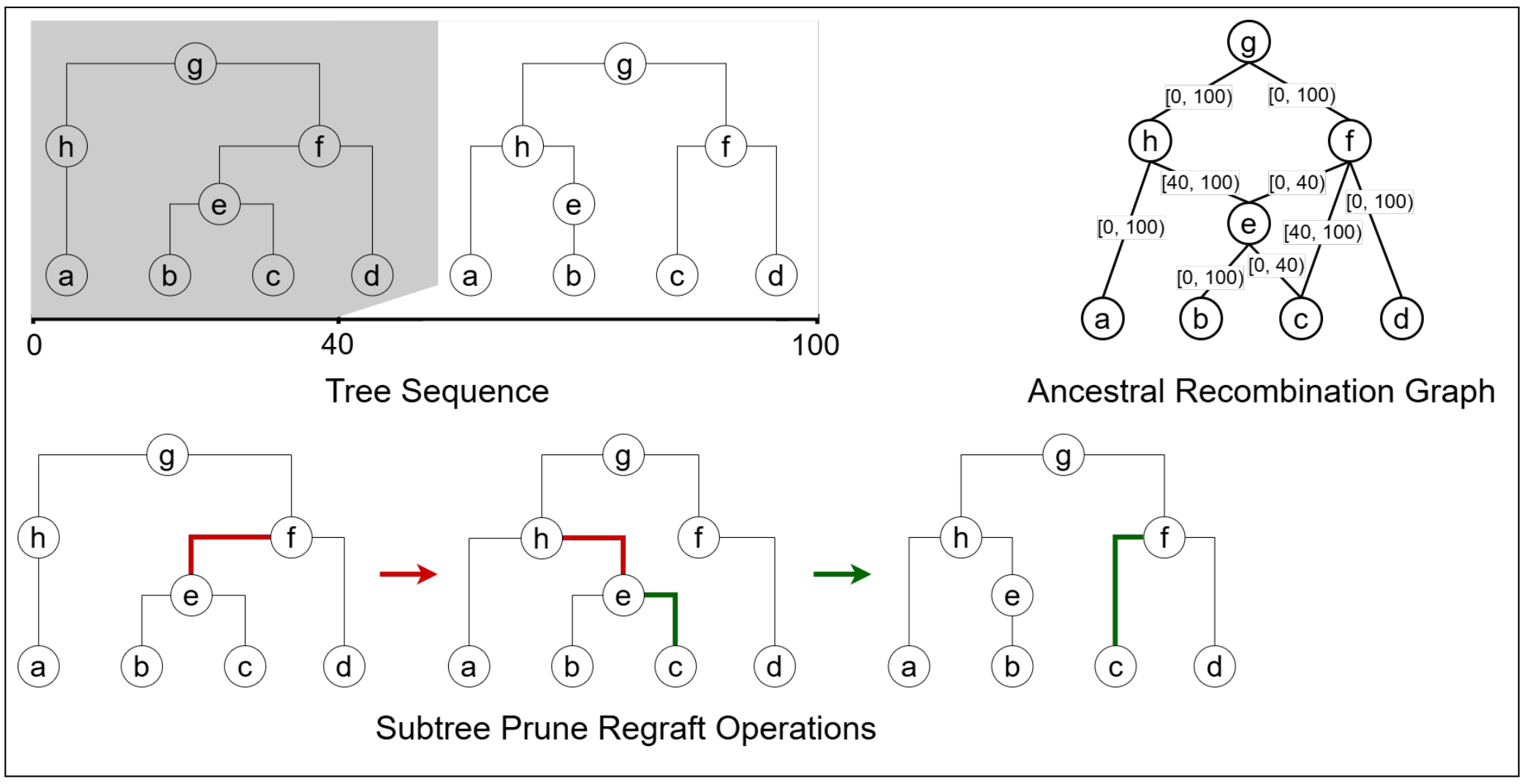

C

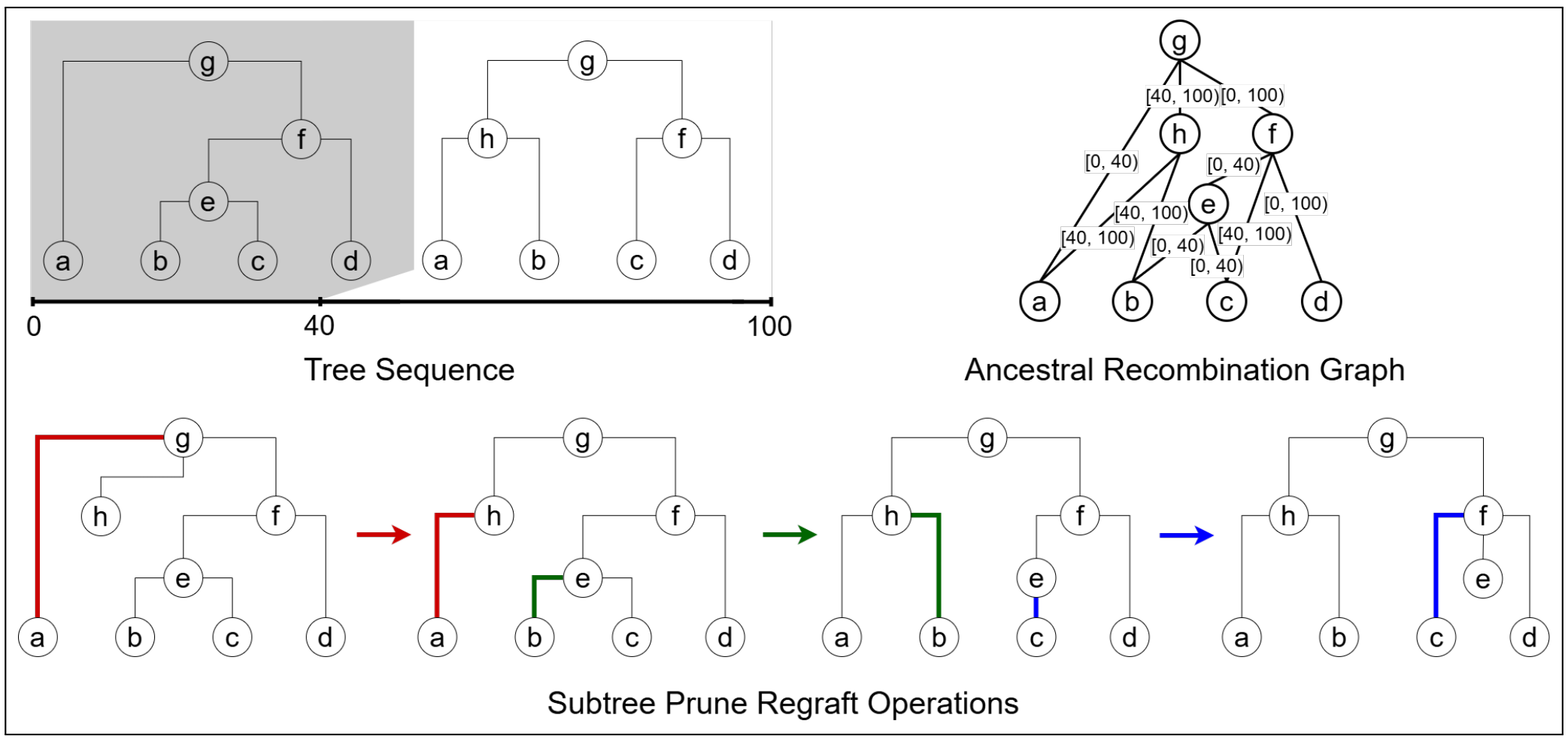

Figure S1. Three tree sequence examples A, B, and C with their corresponding ARGs and SPR operations.

References

1. Baumdicker F, Bisschop G, Goldstein D, Gower G, Ragsdale AP, Tsambos G, et al. Efficient ancestry and mutation simulation with msprime 1.0. Genetics. 2022 03;220(3):iyab229. Available from: https://doi.org/10.1093/genetics/iyab229.
2. Adrion JR, Cole CB, Dukler N, Galloway JG, Gladstein AL, Gower G, et al. A community-maintained standard library of population genetic models. eLife. 2020;9:e54967. Available from: https://doi.org/10.7554/eLife.54967.
3. Lauterbur ME, Cavassim MIA, Gladstein AL, Gower G, Pope NS, Tsambos G, et al. Expanding the stdpopsim species catalog, and lessons learned for realistic genome simulations. eLife. 2023;12:RP84874. Available from: https://doi.org/10.7554/eLife.84874.
4. Gutenkunst RN, Hernandez RD, Williamson SH, Bustamante CD. Inferring the Joint Demographic History of Multiple Populations from Multidimensional SNP Frequency Data. PLoS Genetics. 2009;5(10):1-11. Available from: https://doi.org/10.1371/journal.pgen.1000695.
5. Wong Y, Ignatieva A, Koskela J, Gorjanc G, Wohns AW, Kelleher J. A general and efficient representation of ancestral recombination graphs. Genetics. 2024;228(1):iyae100. Available from: https://doi.org/10.1093/genetics/iyae100.
